# What Is the Correct Name for the New Local Anæsthetic

**Published:** 1885-01

**Authors:** 


### What is the Correct Name for the New Local Anæsthetic?

Dr. D. K. Shute, of Washington, writes to the *Medical Record* (November 29, 1884,) concerning the proper name of the alkaloid of cocaine, which is just now attracting so much attention on account of the wonderful results which are being recorded for it as a local anæsthetic. He says: "It strikes me that the correct technical name for this drug is 'chloride of cocaine.' In an editorial of *The Record* for Nov. 8th, it is stated that 'cocaine unites easily with dilute acids to form crystallizable salts,' therefore if it unites with dilute hydrochloric acid it forms a crystallizable salt, which salt is a 'binary compound' and *not* a 'ternary' one. Modern scientific works on chemistry make the characteristic termination of 'binary compounds' -ide and not -ate. 'Ternary compounds' have the characteristic termination -ate. A scientific chemist never speaks of the 'hydrochlorate of ammonia' as the result of the combination of hydrochloric acid (HCl) and ammonia ($NH_3$); but he calls the resulting compound chloride of ammonia or ammonium chloride ($NH_3HCl$) or, more properly, $NH_4Cl$. It is no more correct to say hydrochlorate of morphine, or hydrochlorate of cocaine, than it is to say hydrochlorate of ammonia. The result of the union of cocaine ($C_{17}H_{21}NO_4$) and hydrochloric acid (HCl) is, as stated above, a 'binary compound.' Therefore, to be correctly specified, it should be written cocaine chloride ($C_{17}H_{22}NO_4Cl$). It is proper to say sulph*ate* or nitr*ate* of cocaine, because the union of nitric and sulphuric acids with cocaine form 'ternary compounds.' I write these few lines in the hope that a drug having such brilliant properties may be dignified by being called by its proper scientific name, viz.: cocaine chloride, chloride of cocaine, or cocaine chloridum."

